# A Thickened Stochastic Fields Approach for Turbulent Combustion Simulation

**DOI:** 10.1007/s10494-018-9954-y

**Published:** 2018-08-14

**Authors:** M. A. Picciani, E. S. Richardson, S. Navarro-Martinez

**Affiliations:** 1 Faculty of Engineering and the Environment, University of Southampton, Southampton, UK; 2 Department of Mechanical Engineering, Imperial College London, London, UK

**Keywords:** Stochastic fields, Probability density function, Premixed combustion, Thickened flame, Turbulent combustion

## Abstract

The Stochastic Fields approach is an effective way to implement transported Probability Density Function modelling into Large Eddy Simulation of turbulent combustion. In premixed turbulent combustion however, thin flame-like structures arise in the solution of the Stochastic Fields equations that require grid spacing much finer than the filter scale used for the Large Eddy Simulation. The conventional approach of using grid spacing equal to the filter scale yields substantial numerical error, whereas using grid spacing much finer than the filter length scale is computationally-unaffordable for most industrially-relevant combustion systems. A Thickened Stochastic Fields approach is developed in this study in order to provide physically-accurate and numerically-converged solutions of the Stochastic Fields equations with reduced compute time. The Thickened Stochastic Fields formulation bridges between the conventional Stochastic Fields and conventional Thickened-Flame approaches depending on the numerical grid spacing utilised. One-dimensional Stochastic Fields simulations of freely-propagating turbulent premixed flames are used in order to obtain criteria for the thickening factor required, as a function of relevant physical and numerical parameters, and to obtain a model for an efficiency function that accounts for the loss of resolved flame surface area caused by applying the thickening transformation to the Stochastic Fields equations. The Thickened Stochastic Fields formulation is tested by performing LES of a laboratory premixed Bunsen flame. The results demonstrate that the Thickened Stochastic Fields method produces accurate predictions even when using a grid spacing equal to the filter scale. The present development therefore facilitates the accurate application of the Stochastic Fields approach to industrially-relevant combustion systems.

## Introduction

Transported Probability Density Function (PDF) modelling of turbulent combustion is advantageous because the composition PDF provides information needed to evaluate the filtered reaction rates required for Large Eddy Simulation (LES), or the mean reaction rates required for Reynolds-averaged simulations. In particular, the PDF approach is valuable for prediction of combustion processes that are sensitive to turbulence-chemistry interactions, including extinction and ignition, and the formation of various pollutants. The Stochastic Fields approach of Valiño [1] has been applied in a number of recent PDF-LES studies because, in contrast with Lagrangian particle PDF formulations, the Stochastic Fields approach guarantees density fields that are continuous in space without the need for special treatment, and it can be solved using the same Eulerian numerical implementation as the LES momentum equations. Stochastic Fields PDF-LES has been used to model both non-premixed [2] and premixed combustion [3]. However, in Stochastic Fields simulation of premixed combustion, flame-like structures arise that may be thinner than the LES filter length scale [4]. In order to solve the Stochastic Fields equations accurately it is then necessary to have grid spacing finer than the filter length scale substantially adding to the computational time required for the Stochastic Fields simulation. Conversely, following the conventional practice of setting the LES filter length scale equal to the numerical grid spacing can lead to substantial numerical error for two reasons [4]. First, numerical diffusion caused by under-resolution changes the local propagation speed of the reaction fronts in the Stochastic Fields solution. Second, wrinkling of the reaction fronts by resolved turbulence is reduced because the numerical diffusion increases the thickness of the fronts. Having identified the difficulty and importance of spatially-resolved Stochastic Fields solutions for premixed combustion in Ref. [4], the present contribution seeks to develop a practical approach to alleviate the demanding resolution requirements.

The Thickened Flame approach [5, 6] has been introduced as a means to ensure accurate numerical resolution of premixed reaction fronts in LES. In the Thickened Flame approach, the governing equations for composition and energy are modified in order to yield thicker reaction fronts that can be resolved accurately on a given numerical grid, and an *efficiency function* model is employed to compensate for the reduction of flame wrinkling that results from the artificial thickening. Importantly, the Thickened Flame approach removes uncharacterised numerical errors that depend both on the numerical grid and numerical methods employed. Instead, the quality of the predictions depends on the accuracy of the efficiency function modelling employed. The efficiency function model should account for effects of un-resolved flame wrinkling on the overall burning rate, but it lacks the more general ability of PDF approaches to describe turbulence-chemistry interactions. The objective of this paper is to set out a new approach for Stochastic Fields-PDF simulation that uses artificial thickening to ensure accurate numerical solution on any given numerical grid. The Thickened Stochastic Fields (TSF) approach retains at least some of the ability of PDF methods to describe turbulence-chemistry interactions and recovers the standard Stochastic Fields formulation when the numerical resolution is sufficient.

## Development of the Thickened Stochastic Fields Approach

The Thickened Stochastic Fields approach is best introduced by first reviewing the formulation of the Thickened Flame approach. Consider a scalar transport equation describing reactive flow:

1 $$ \rho\frac{\partial \mathbf{Y}}{\partial t}=-\rho {u_{j}}\frac{\partial \mathbf{Y}}{\partial x_{j}}+\frac{\partial}{\partial x_{j}}\left( \rho D \frac{\partial \mathbf{Y}}{\partial x_{j}}\right)+\dot{\mathbf{\omega}}(\mathbf{Y}), $$

where **Y** is the vector of species mass fractions and enthalpy, *u*_*j*_ is the j^*th*^ component of the velocity vector, *D* is the laminar diffusivity (assumed equal for all species), and $\dot {\mathbf {\omega }}$ is the vector of chemical source terms.

### The thickened flame model

The Thickened Flame equation [5] is obtained by applying the transformation **x**′ = *F***x** and *t*′ = *Ft*/*E* [5] to Eq. 1,

2 $$ \rho\frac{\partial \mathbf{Y}}{\partial t^{\prime}}=-\rho Ev_{j}\frac{\partial \mathbf{Y}}{\partial x_{j}^{\prime}}+\frac{\partial}{\partial x_{j}^{\prime}}\left( \rho DEF \frac{\partial \mathbf{Y}}{\partial x_{j}^{\prime}}\right)+\frac{E}{F}\dot{\mathbf{\omega}}(\mathbf{Y}), $$

where the convection velocity **v** is given by the solution of the similarly-transformed Navier-Stokes equations [7]. In most previous applications of the Thickened Flame approach, following Refs. [5, 6], the thickened scalar transport equation Eq. 2 has been coupled with unthickened LES making the assumption that *E***v** is equal to the resolved velocity from the LES simulation $\tilde {\mathbf {u}}$. The effect of thickening factor *F* and efficiency function *E* can be understood by considering the solution of a stationary freely-propagating planar premixed flame (*∂*/*∂t*′ = *∂*/*∂t* = 0). The flame thickness given by Eq. 2 is thickened by the factor *F* and the propagation speed is faster by a factor *E* compared respectively to the flame thickness *δ*_*L*_ and flame speed *S*_*L*_ given by solution of Eq. 1.

Thickening the species transport equations by factor *F* with *E* = 1 has the attractive feature that numerical resolution requirements are reduced while laminar flame speeds are unaffected. The turbulent flame speed, however, depends on the increase in flame surface area caused by wrinkling of the flame front. The amount of wrinkling depends (at least) on the ratio of turbulent velocity fluctuations to the laminar flame speed *u*′/*S*_*L*_, and the ratio of the turbulence length scales to the laminar flame thickness, *L*_*T*_/*δ*_*L*_. Thickening the flame front to *Fδ*_*L*_ reduces the degree to which the turbulence will wrinkle the flame. The efficiency function *E* can then be used as a correction factor that increases the local propagation speed in order to compensate for the loss of resolved flame surface area resulting from application of the thickening transformation.

The local propagation speed of the reaction-front resolved by the LES simulation is described as the sub-filter turbulent flame speed *S*_*T*Δ_ [6]. The ratio of the sub-filter turbulent flame speed *S*_*T*Δ_ and the laminar flame speed *S*_*L*_ is assumed to be equal to wrinkling factor Ξ, which is equal to the projection of the sub-filter scale flame area in the direction of propagation,

3 $$ \frac{S_{T{\Delta}}}{S_{L}}=\frac{A_{sfs}}{{\Delta}^{2}}={\Xi}_{{\Delta}}. $$

**Fig. 1:**
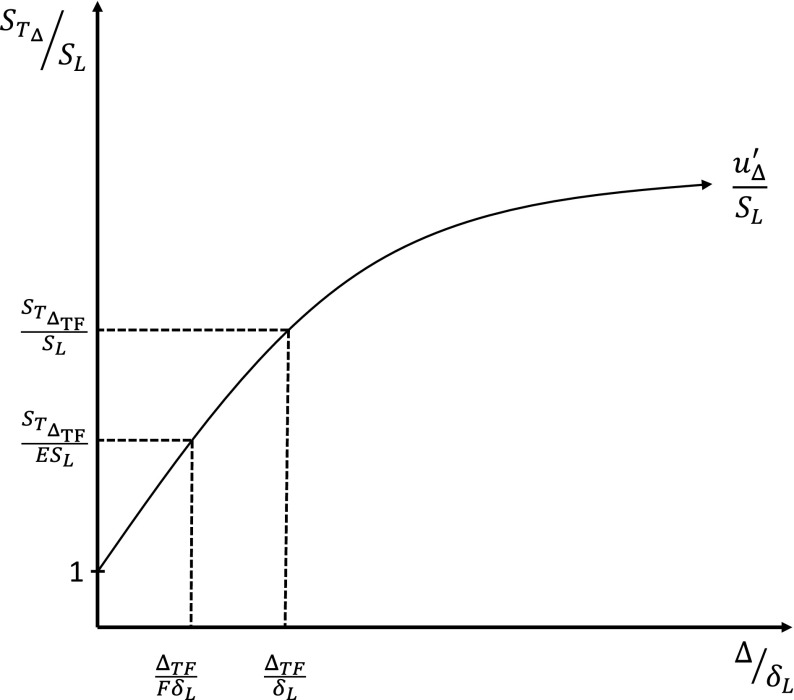
The dependence of wrinkling factor *S*_*T*Δ_/*S*_*L*_ on Δ/*δ*_*L*_ and $u^{\prime }_{{\Delta }}/S_{L}$, indicating the reduction in Ξ_Δ_ due to thickening by factor *F*

Modelling for the sub-filter flame wrinkling in the context of Thickened Flame modelling has been proposed initially by Colin et al. [5] and Charlette et al. [6] as functions of the non-dimensional sub-filter velocity fluctuations *u*′/*S*_*L*_ and the non-dimensional filter size Δ_*TF*_/*δ*_*L*_, where Δ_*TF*_ = *Fδ*_*L*_ is the effective filter scale in the Thickened Flame model. In general, the effective filter scale Δ_*TF*_ implied by the Thickened Flame model can be different from the filter length scale Δ used in modelling of the LES momentum or Stochastic Fields equations. Thickening the flame front by factor *F* reduces the non-dimensional filter size to Δ/*Fδ*_*L*_, resulting in a reduction in the sub-filter turbulent flame speed by factor 1/*E* as illustrated in Fig. 1. The efficiency function *E* is defined in Ref. [5] as the ratio of the wrinkling factor in the thickened and unthickened flames,

4 $$ E_{TF}=\frac{{\Xi}_{{\Delta}}\left( u^{\prime}_{{\Delta},{TF}}/S_{L},{{\Delta}_{TF}}/{\delta_{L}}\right)}{{\Xi}_{{\Delta}}\left( {u^{\prime}_{{\Delta},{TF}}}/{S_{L}},{{\Delta}_{TF}}/{F\delta_{L}}\right)}. $$

### The thickened stochastic fields model

The unmodified Stochastic Fields equation is given by Valiño et al. [8] as,

5 $$\begin{array}{@{}rcl@{}} \overline{\rho}\text{d}{\zeta}_{(i)}&=&-\overline{\rho}\widetilde{u_{j}}\cdot\frac{\partial{\zeta}_{(i)}}{\partial x_{j}}\text{d}t+\frac{\partial}{\partial x_{j}}\left( \overline{\rho} (D+D_{T})\frac{\partial{{\zeta}_{(i)}}}{\partial x_{j}}\right)\text{d}t +\overline{\rho}\sqrt{2D_{T}}\frac{\partial{\zeta}_{(i)}}{\partial x_{j}}d{W_{j}}_{(i)} \\ &&- \frac{\overline{\rho}}{\tau_{T}}\left( {\zeta}_{(i)}-\widetilde{{\zeta}}\right)\text{d}t+\overline{\rho}\dot{{\omega}}({\zeta}_{(i)})\text{d}t. \end{array} $$

where *ζ*_(*i*)_(**x**, *t*) is the value of the composition vector on the *i*^th^ field. The terms on the right hand side represent the evolution of the stochastic field composition due to advection by the mean (or resolved) velocity; spatial diffusion by molecular *D* and turbulent *D*_*T*_ diffusivities; turbulent advection of fields relative to one another as modelled by a Wiener process where d*W*_*j*__(*i*)_ is the component in the *j*^th^ direction of a normally-distributed Markovian random increment with zero mean and variance equal to the time step *dt*; unresolved scalar dissipation processes modelled by interaction by exchange with the mean (IEM) [9] with dissipation time scale $\tau _{T}^{-1}=C_{\phi {\Delta }}(D+D_{T})/{\Delta }^{2}$; and the vector of chemical reaction source terms $ {\dot {\omega }}( {\zeta }_{(i)})$.

The Thickened Stochastic Fields equation is obtained by applying to Eq. 5, the same transformation that produces the Thickened Flame model in Section 2.1: **x**′ = *F***x**, *t*′ = *Ft*/*E*, and, since the Wiener increment vector **dW** has dimension $\sqrt {t}$, $\mathbf {dW}^{\prime }=\sqrt {F/E}\mathbf {dW}$:

6 $$\begin{array}{@{}rcl@{}} \overline{\rho}\text{d}{\zeta}_{(i)}&=&-\overline{\rho}E\widetilde{v_{j}}\cdot\frac{\partial{\zeta}_{(i)}}{\partial x_{j}^{\prime}}\text{d}t^{\prime}+\frac{\partial}{\partial x_{j}^{\prime}}\left( \overline{\rho} (D+D^{\prime}_{T})EF\frac{\partial}{\partial x_{j}^{\prime}}{{\zeta}_{(i)}}\right)\text{d}t^{\prime}\\ &&+ \overline{\rho}\sqrt{2D^{\prime}_{T}EF}\frac{\partial{\zeta}_{(i)}}{\partial x_{j}^{\prime}}d{W_{j}^{\prime}}_{(i)} -\frac{\overline{\rho}E\left( {\zeta}_{(i)}-\widetilde{{\zeta}}\right)}{F\tau^{\prime}_{T}}\text{d}t^{\prime}+\frac{\overline{\rho}E\dot{{\omega}}({\zeta}_{(i)})}{F}\text{d}t^{\prime}. \end{array} $$

In principle the convection velocity $\tilde {\mathbf {v}}$, turbulent diffusivity $D^{\prime }_{T}$, and dissipation time scale $\tau ^{\prime }_{T}$ come from solution of similarly-transformed LES momentum equations. However if, following Colin et al. [5], the thickened scalar equations are coupled with unthickened LES momentum equations then the velocity $\tilde {\mathbf {u}}$ and turbulent diffusivity *D*_*T*_ from the unthickened LES should be scaled as: $\tilde {\mathbf {v}}=\tilde {\mathbf {u}}/E$ and $D^{\prime }_{T}=D_{T}/{EF}$. The turbulence timescale to be used in Eq. 6 is then $\tau _{T}^{\prime -1}=C_{\phi {\Delta }}(DEF+D_{T})/(F{\Delta })^{2}$.

The transformation of the Stochastic Fields equation has the effect that the solution for a steady-state planar freely-propagating turbulent flame modelled by Eq. 6 is thickened by factor *F* and the propagation speed is increased by factor *E* relative to the solution of Eq. 5. The thickening factor *F* can therefore be set in order to obtain satisfactory numerical resolution on a particular computational grid. The efficiency function *E* should then be set in order to account for the reduction in resolved flame surface area that results from thickening of the Stochastic Fields equation.

### The efficiency function

The specification of the efficiency function for the Thickened Stochastic Fields model relates to the wrinkling of the reaction fronts in the Stochastic Fields solution, rather than the wrinkling of physical flames considered in the conventional Thickened Flame model. The characteristic thickness $\delta _{c^{*}}$, and propagation speed $S_{c^{*}}$, of the reaction fronts in the Stochastic Fields solution are in general different from the thickness and speed of the corresponding laminar flame. However, the wrinkling dynamics of the reaction fronts are assumed to be governed by the same function, Ξ_Δ_. This assumption is justified because in both Thickened Flame and Thickened Stochastic Fields, the wrinkling dynamics of reaction fronts in the flamelet regime are dominated by the combination of resolved convection, sub-filter turbulent transport, diffusion, and reaction processes. The efficiency function for the Thickened Stochastic Fields model (Eq. 6) is then given by,

7 $$ E_{TSF}=\frac{{\Xi}_{{\Delta}}\left( {u^{\prime}_{{\Delta},{TSF}}}/{S_{c^{*}}},{{\Delta}_{TSF}}/{\delta_{c^{*}}}\right)}{{\Xi}_{{\Delta}}\left( {u^{\prime}_{{\Delta},{TSF}}}/{S_{c^{*}}},{{\Delta}_{TSF}}/{F\delta_{c^{*}}}\right)}, $$

where the effective filter scale of the thickened stochastic fields is ${\Delta }_{TSF}=F\delta _{c^{*}}$. In general Δ_*TSF*_ can be different from the filter scale Δ used to evaluate the model for the turbulent diffusivity in Eq. 6.

Previous studies have developed models for the function Ξ_Δ_ on the basis of theory and empirical information from direct numerical simulations and laboratory measurements of flame response [5, 6]. The purpose of the TSF approach however is to provide simulation results that maintain the same flame propagation speeds as the underlying Stochastic Fields modelling when the computational grid spacing is increased. The modelling for Ξ_Δ_ should not seek to improve the agreement between the Stochastic Fields model and DNS or experiment, rather it should fit to predictions of the underlying unthickened Stochastic Fields model. Improving the physical accuracy of the underlying Stochastic Fields modelling is outside the scope of the present study. The functional dependence of the wrinkling factor on the filter-scale turbulence properties ${\Xi }_{{\Delta }}(u^{\prime }_{{\Delta }}/S_{L},{\Delta }/\delta _{L})$ is therefore obtained from Stochastic Fields simulations across a range of conditions. The set up of one-dimensional Stochastic Fields simulations in order to obtain data for $\delta _{c^{*}}/\delta _{L}$ and $S_{c^{*}}/S_{L}$ is presented in the next Section.

### Determination of wrinkling factor from 1D stochastic fields simulations

The dependence of the sub-filter scale turbulent flame speed and reaction-front thickness on filter-scale turbulence properties is evaluated in a one-dimensional Stochastic Fields simulation of a freely-propagating planar turbulent flame. The one-dimensional approach neglects the effects that curvature and bulk strain have on the local propagation of reaction-fronts in Stochastic Fields LES of premixed combustion, but has the advantage that the simulations are computationally inexpensive compared to three-dimensional LES and still represent the transport processes normal to the resolved reaction front that dominate the dynamics of the resolved reaction front.

#### One-dimensional closures

The filter-scale turbulence properties are specified by the filter length scale Δ and the corresponding sub-filter scale velocity fluctuation $u_{{\Delta }}^{\prime }$. The turbulent diffusivity required in Eq. 5 is modelled as

8 $$ D_{T}=C_{\mu{\Delta}}u_{{\Delta}}^{\prime}{\Delta}, $$

with *C*_*μ*Δ_ = 0.09. The turbulent mixing frequency is modelled by

9 $$ \tau_{T} = \frac{{\Delta}^{2}}{C_{\phi{\Delta}}(D+D_{T})} $$

with model coefficient *C*_*ϕ*Δ_ = 2.0 (*μ*_*L*_/*μ*_*T*_ + 1) [10].

Closure for the sub-filter turbulent diffusivity in Eq. 8 cannot be obtained by traditional means, such as with the Smagorinsky model, due to the one-dimensional nature of the simulations. Instead, a model for the variation of the sub-filter scale velocity rms is determined from a scaling analysis, using the assumption that the sub-filter dissipation rate is independent of filter scale for filter length scales in the inertial range. The sub-filter velocity fluctuations are then related to the combustion regime, characterised by the Karlovitz number, *Ka* = [(*u*′/*S*_*L*_)^3^(*δ*_*L*_/*L*_*T*_)]^1/2^. The result of the scaling analysis is that the sub-filter velocity fluctuations scale according to

10 $$ u_{{\Delta}}^{\prime}=S_{L}\text{Ka}_{{\Delta}}^{2/3}\left( \frac{{\Delta}}{\delta_{L}}\right)^{1/3}, $$

where *Ka*_Δ_ the filter scale Karlovitz number. With the assumption of constant dissipation in the inertial range, the Karlovitz number is scale invariant [6] and the the filter scale Karlovitz number can be expressed as the integral scale Karlovitz number; Ka_Δ_ = Ka.

#### Evaluation of *E*_*TSF*_

$S_{c^{*}}/S_{L}$ and $\delta _{c^{*}}/\delta _{L}$ required in Eq. 7 are evaluated over a wide range of range of $u^{\prime }_{{\Delta }}/S_{L}$ and Δ/*δ*_*L*_ corresponding to a wide range of premixed combustion regimes. The numerical methods and thermo-chemical models employed are described in the subsequent section. 512 Stochastic Fields are used for the one-dimensional simulations with uniform computational grid spacing selected to ensure at least 16 points within the reaction fronts in each case.

The average consumption speed of the reaction front of the individual stochastic fields is evaluated by calculating the overall consumption speed of the ensemble average of the stochastic fields. In this statistically-stationary case, the overall consumption speed of the ensemble average of the stochastic fields is necessarily equal to the averaged consumption speed of the individual stochastic fields ($S_{c^{*}}=S_{T{\Delta }}$). The consumption speed is evaluated through

11 $$ S_{T{\Delta}}=\frac{1}{\rho_{u}Y_{f}A}{\int}_{V}\frac{1}{N_{s}}\sum\limits_{i = 1}^{N_{s}}\dot{\omega}_{(i)}\text{dV}, $$

where *ρ*_*u*_ is the unburnt gas density, *Y*_*f*_ the mass fraction of fuel in the premixed reactants, *A* the domain cross sectional area, *N*_*s*_ represents the number of Stochastic Fields, and $\dot {\omega }_{(i)}$ the instantaneous reaction rate on field *i*. Due to the stochastic nature of the consumption speed given by Eq. 5, the values reported are those averaged over time.

**Fig. 2:**
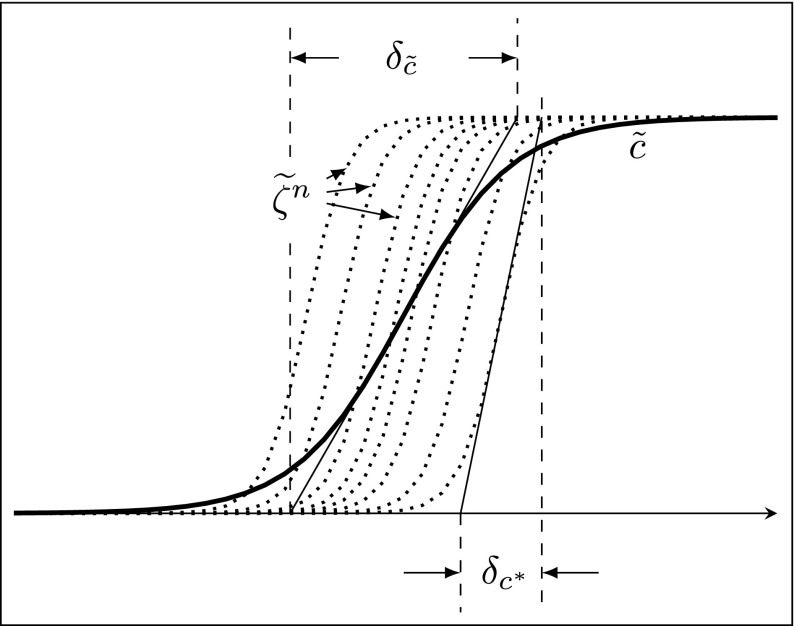
A schematic diagram showing the thickness ($\delta _{c^{*}}$) of the individual stochastic fields progress variable profiles (dashed lines) and the thickness ($\delta _{\tilde {c}}$) of the resolved flame progress variable profile (solid line)

The average thicknesses of the individual stochastic fields (illustrated in Fig. 2) is evaluated as

12 $$ \left\langle\delta_{c^{*}}\right\rangle=\frac{1}{N_{s}}\sum\limits_{i = 1}^{N_{s}}\frac{1}{|\nabla{\zeta_{(i)}}|_{max}}. $$

Similar to the consumption speed, the average stochastic field thickness reported is also the time averaged value.

The resultant data sets are approximated by fitting power-law functions of $u^{\prime }_{{\Delta }}/S_{L}$ and Δ/*δ*_*L*_ in the general form employed by Charlette et al. [6],

13 $$ f_{i}(u^{\prime}_{{\Delta}}/S_{L},{\Delta}/\delta_{L})=\left( 1+A_{i}\left( \frac{u^{\prime}_{{\Delta}}}{S_{L}}\right)^{a_{i}}\left( \frac{{\Delta}}{\delta_{L}}\right)^{b_{i}}\right)^{\beta_{i}}. $$

**Fig. 3:**
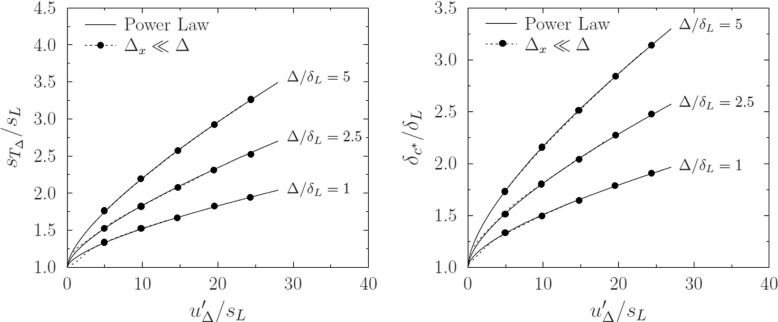
Wrinkling factor $S_{T_{{\Delta }}}/S_{L}$ (left) and non-dimensional reaction front thickness $\delta _{c^{*}}/\delta _{L}$ (right) versus sub-filter turbulence intensity $u^{\prime }_{{\Delta }}/S_{L}$ for well-resolved Stochastic Fields (${\Delta }_{x}\ll {\Delta }$), Stochastic Fields simulation with ${\Delta }_{x}={\Delta }$, and the curve fit to the well resolved data given by Eq. 13

The ranges of $u^{\prime }_{{\Delta }}/S_{L}$ and Δ/*δ*_*L*_ used to fit the coefficients in Eq. 13 correspond to ranges of Karlovitz number Ka ∈ (0.5 − 50) and filter length scale ratios Δ/*δ*_*L*_ ∈ (1 − 5) that are representative of practical LES simulations of premixed combustion in internal combustion engines and gas turbines. A least squares fit to the data yields [*A*_*S*_, *a*_*S*_, *b*_*S*_, *β*_*S*_] = [0.083, 0.627, 0.48, 1.4] for $f_{S}=S_{c^{*}}/S_{L}$ and [*A*_*δ*_, *a*_*δ*_, *b*_*δ*_, *β*_*δ*_] = [0.081, 0.6, 0.47, 1.48] for $f_{\delta }=\delta _{c^{*}}/\delta _{L}$. The curve-fits give excellent agreement across the relevant parameter space, as shown in Fig. 3.

With an appropriate model for the dependence of the sub-filter scale turbulence velocity fluctuation $u^{\prime }_{{\Delta }}$ on the filter length scale, and with knowledge of the laminar flame speed and thickness, the Thickened Stochastic Fields Efficiency Function *E*_*TSF*_ can be evaluated through the following steps:

1. Evaluate $u^{\prime }_{{\Delta }}$ for the Stochastic Fields filter scale Δ;
2. Evaluate $\delta _{c^{*}}/\delta _{L}$ and $S_{c^{*}}/S_{L}$ corresponding to $u^{\prime }_{{\Delta }}/S_{L}$ and Δ/*δ*_*L*_ using Eq. 13;
3. Evaluate $F=n{\Delta }_{x}/\delta _{c^{*}}$ and Δ_*TSF*_ = *n*Δ_*x*_, where *n* is the minimum number (e.g. 5-7) of grid spacings Δ_*x*_ required within the reaction-front thickness $\delta _{c^{*}}$;
4. Calculate $u^{\prime }_{{\Delta },{TSF}}$ for the effective Thickened Stochastic Fields filter scale Δ_*TSF*_;
5. Evaluate Eq. 7 for *E*_*TSF*_ using the power law curve fit Eq. 13 for the wrinkling factors.

## Turbulent Premixed Bunsen Flame LES

**Fig. 4:**
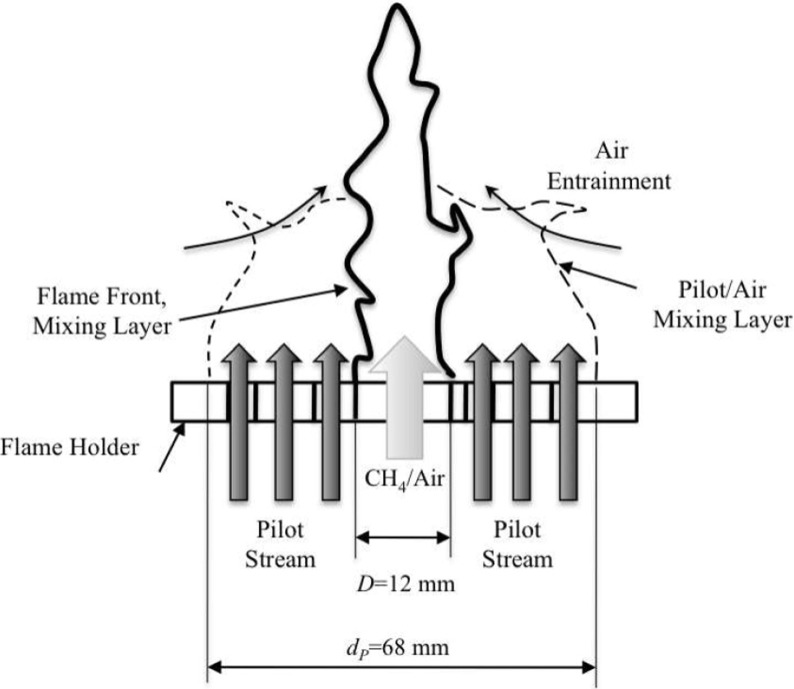
Schematic of the burner of Chen et al. [11]. Image obtained from [3]

The turbulent premixed Bunsen flame of Chen et al. [11] is illustrated schematically in Fig. 4. The F3 turbulent premixed Bunsen flame described by Chen et al. [11] is simulated using Stochastic Fields-LES and the Thickened Stochastic Fields-LES. The numerical set-up is identical to previous Stochastic Fields simulations in Ref. [4], however the formulation is repeated in Sections 3.1 and 3.2 for completeness. The flame is characterised by Karlovitz numbers of order unity, indicating that combustion takes place across the flamelet and thin reaction zone regimes. Since it is difficult to infer information about these flamelet structures from a one-point PDF, this test case presents a challenge for PDF methods. The flame has simple boundary conditions and has served as the basis for numerous investigations of PDF modelling for turbulent premixed combustion [3, 12–15].

A 12 mm diameter nozzle delivers a turbulent jet of 300 K, stoichiometric methane-air with bulk velocity 30 ms^− 1^. The flame is stabilised by a ring of stoichiometric methane-air pilot flames surrounding the nozzle with 68 mm outer diameter and bulk velocity 1.32 ms^− 1^. Further downstream the flame entrains 300 K air at 1 atm from a quiescent laboratory environment. Profiles of mean and rms velocity fluctuations are reported in [11] and are used to set the inflow profiles for the simulation.

The largest source of uncertainty with this particular test case is the pilot temperature due to heat loss between the pilot flame, the burner, and the environment. Different numerical simulations of this series flame have used varying values of the pilot flame temperature, however in this work, it has been estimated as in previous LES studies [3] as 1785 K. Due to the use of simplified chemistry and the omission of certain chemical species, the pilot composition was modified from that outlined in [11] and is given in Table 1.

**Table 1:** Pilot stream composition

Species	Chen et al. [11]	Current work
$Y_{O_{2}}$	5.00E-4	5.00E-4
$Y_{H_{2}O}$	0.1236	0.1236
$Y_{CO_{2}}$	0.15	0.15
*Y*_*CO*_	7.800E-4	–
$Y_{H_{2}}$	3.00E-5	–
*Y*_*OH*_	1.20E-4	–
$Y_{N_{2}}$	0.7247	0.7259

The spatially-filtered continuity and momentum equations [3] are closed with the constant-coefficient Smagorinsky model for the sub-filter scale turbulent stresses [16], with Smagorinsky constant equal to 0.09. The turbulent diffusivity required in Eq. 5 is modelled assuming turbulent Schmidt number equal to 0.7 and the dissipation time scale is modelled by Eq. 9 with model coefficient *C*_*ϕ*Δ_ once again scaled as proposed in [10].

Calculation of the sub-filter scale velocity fluctuations required for the evaluation of *E*_*TSF*_ (as outlined in Sec. 2.4.2), follows the procedure used in the traditional Artificially Thickened Flame approach derived in [5]. The sub-filter scale velocity fluctuations in the Thickened Stochastic Fields approach are evaluated as

14 $$ u^{\prime}_{{\Delta}}=c_{2}{\Delta}^{3}\lvert \nabla\times(\nabla^{2}(\tilde{\mathbf{u}}))\rvert, $$

where $\tilde {\mathbf {u}}$ is the resolved velocity corresponding to filter scale Δ and *c*_2_ = 2.0 is a model constant [5]. The use of this relation ensures zero velocity fluctuation in the limit of a laminar flame, which also enforces the correct asymptotic behaviour of the Thickened Stochastic Fields framework in this limit.

### Thermo-chemical models

The premixed combustion kinetics are modelled using a one-step reaction model for methane-air flames,

15 $$ CH_{4}+ 2O_{2}\rightarrow CO_{2}+ 2H_{2}O. $$

The fuel reaction rate is modelled by the Arrhenius law,

16 $$ \dot{\omega}_{CH_{4}}=A\cdot\left( \frac{\rho Y_{CH_{4}}}{M_{CH_{4}}} \right)^{n_{CH_{4}}}\left( \frac{\rho Y_{O_{2}}}{M_{O_{2}}} \right)^{n_{O_{2}}}\exp\left( -\frac{E_{a}}{RT} \right), $$

where *T*, $Y_{CH_{4}}$, $Y_{O_{2}}$, $M_{CH_{4}}$, $M_{O_{2}}$ and *R* denote temperature, fuel and oxygen mass fractions, corresponding molar masses and the universal gas constant, respectively. The pre-exponential factor, the activation energy and the model exponents are *A* = 1.1 × 10^10^ (cgs), *E*_*a*_ = 20,000 cal/mol, $n_{CH_{4}}= 1.0$ and $n_{O_{2}}= 0.5$. The use of such simple chemical modelling is justified by the focus of the present study on evaluation of the numerical resolution requirements of the Stochastic Fields approach, rather than assessing the physical accuracy of the Stochastic Fields approach.

Temperature dependent properties are modelled with NASA polynomials and, due to the inherent unity Lewis number assumption in the Stochastic Fields formulation, the Schmidt and Prandtl numbers are both set equal to 0.7, while the mixture kinematic viscosity is modelled with Wilkes law. These assumptions lead to a laminar flame speed *S*_*L*_ = 0.38 ms^− 1^, a thermal thickness of 0.408 mm and a burnt gas adiabatic temperature *T*_*b*_ = 2328 K in atmospheric stoichiometric conditions.

### Numerical implementation and simulation setup

The Stochastic Fields equation is implemented within the block-structured BOFFIN computational fluid dynamics code [3, 17]. The code is a second order accurate finite volume method based on fully implicit low-Mach-number formulation using a staggered storage arrangement. For the momentum equation convection term, an energy conserving discritization scheme is used and all other spatial derivatives are approximated by standard second order central differences. A TVD scheme is used for the convection terms in the scalar conservation equations. The stochastic field equations are solved using a weak first order temporal approximation with accuracy $\mathcal {O}\left (\sqrt {{\Delta }{t}}\right )$ based on the Euler–Maruyama scheme [18]. The Wiener process is approximated by time-step increments $dt^{1/2}{\eta ^{n}_{i}}$ where ${\eta ^{n}_{i}}$ is a {− 1, 1} dichotomic random vector [19]. The chemical source terms are solved using a Newton method-based stiff solver.

The turbulent Bunsen flame is simulated using two different computational grids: a fine grid characterised by 0.5 mm grid spacing at the inlet, and a coarse grid characterised by 1.0 mm grid spacing at the inlet. Both grids are Cartesian, with a region of uniform transverse grid spacing around the inlet with transverse extent equal to twice the nozzle diameter. The axial grid spacing increases linearly in the axial direction. Sixteen stochastic fields are used in the three-dimensional simulations. The computational time step for the respective cases are 4.6*μs* and 2.3*μs*. The turbulent inflow is modelled with the digital filter based method of Klein et al. [20] using the mean and rms velocity profiles from [11].

### Flame sensor

Artificial thickening of the scalar equations is typically necessary only in regions of the flow containing thin reaction fronts. Thickening in regions where it is not required leads to an unnecessary loss of simulation fidelity, for example by over-predicting the rate of fuel-air premixing in partially-premixed combustion systems. To overcome this, Durand et al. [21] introduced dynamic thickening by a flame sensor Ω to remove the effects of thickening away from reaction fronts. This flame sensor is given by

17 $$ {\Omega}= 16\left( {c}\left( 1-{c}\right)\right)^{2}, $$

where *c* is a relevant progress variable equal to zero in the reactants and unity in the products. Ω > 0 indicates the presence of a flame front and Ω = 0 its absence. The thickening factor in the scalar transport equations are then rewritten as

18 $$ F = 1+(F_{0}-1){\Omega}, $$

where *F*_0_ is the thickening factor determined as a function of the grid spacing and the desired number of grid points within the reaction front.

**Fig. 5:**
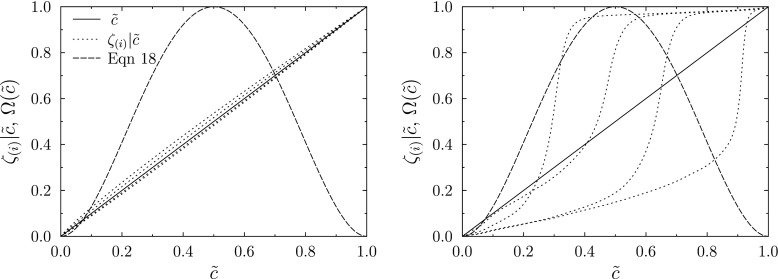
Progress variable on selected stochastic fields for one-dimensional stochastic field simulations with *Ka* = 50, Δ/*δ*_*L*_ = 1 (left) and *Ka* = 0.5, Δ/*δ*_*L*_ = 5 (right). The value of the flame sensor given by Eq. 17 is also shown

A flame sensor can also be applied in the Thickened Stochastic Fields approach. However, evaluating Eq. 17 using the filtered progress variable obtained by ensemble averaging the stochastic fields causes the individual stochastic fields’ reaction fronts to be thickened to different extents, depending on their position in the resolved flame front. This is illustrated in Fig. 5, which presents the variation of the progress variable of selected individual stochastic fields *ζ*_(*i*)_ with respect to the ensemble averaged progress variable $\tilde {c}$ for a one-dimensional flame with low sub-filter variance (*Ka* = 50 and Δ/*δ*_*L*_ = 1) and a flame with high sub-filter variance (*Ka* = 0.5 and Δ/*δ*_*L*_ = 5). The flame sensor value given by Eq. 17 is also presented. Thickening of the stochastic fields is most necessary in the range 0.5 < *ζ*_(*i*)_ < 0.8 where the reaction rate is greatest. Figure 5 shows that stochastic fields with their reaction fronts near to the leading or trailing edge of the resolved flame front might not be thickened to the extent intended.

In order to ensure sufficient thickening of every field, the flame sensor can be evaluated for every field

19 $$ {\Omega}_{(i)}= 16\zeta_{(i)}^{2}\left( 1-\zeta_{(i)}\right)^{2}, $$

and the maximum value then applied to all fields

20 $$ {\Omega}(\mathbf{x},t)={\max}({\Omega}_{(i)}(\mathbf{x},t)). $$

This approach yields the desirable behaviour that Eq. 17 is recovered in the limit of negligible sub-filter progress variable variance.

To ensure a smooth variation of Ω through the resolved flame front in simulations with a limited number of stochastic fields, Eq. 19 is modified to broaden the region of influence of each field according to

21 $$ {\Omega}_{(i)}=\frac{\tanh\left[\beta\left( 16c_{(i)}^{2}\left( 1-c_{(i)}\right)^{2}\right)\right]}{\tanh{\beta}}, $$

where *β* is a parameter greater than unity that broadens the thickening zone for each field and determines how quickly the sensor approaches unity for *c* > 0 and *c* < 1. This broadening of the thickening zone of the individual fields increases the overlap between regions of large Ω_(*i*)_ and together with Eq. 20, setting *β* = 5 provided a smooth variation of Ω through the resolved flame front in the simulations presented below.

## Results and Discussion

### Estimated resolution requirements

The stochastic field reaction front thicknesses reported in Fig. 3 and approximated by Eq. 13 may be used to estimate the resolution requirements for Stochastic Field LES. In the case of the Bunsen flame simulated in this study [11], the thermal thickness of a laminar stoichiometric methane-air flame is 0.41*mm* (using the one-step chemistry model described above), the filter length scale selected is 1*mm*, and the Karlovitz number is reported to range between 1 and 10 [11]. These Karlovitz numbers (corresponding to $u^{\prime }_{{\Delta }}/S_{L}= 1.3$ and 6.2 respectively) lead to the prediction that $\delta _{c^{*}}/\delta _{L} = 1.3 - 1.7$, given Δ = 1*mm*. Imposing a minimum requirement of 5 grid spacings within the stochastic field reaction front thickness $\delta _{c^{*}}$ then dictates a maximum grid spacing of 0.11 - 0.14 mm for fully-resolved Stochastic Fields simulation. Compared to the usual practice of setting the grid spacing equal to the LES filter scale, this estimate suggests a numerically-resolved stochastic field solution would require a grid spacing of 11-14% of the filter scale, implying 360-750 times more grid points in a three-dimensional simulation. The resolution requirements of the Stochastic Fields approach may be even more daunting in simulation of industrial combustion systems for which the ratio of filter length scale to laminar flame thickness might need to be an order of magnitude greater than in the laboratory Bunsen flame example. The need for sub-filter scale resolution around the stochastic field reaction fronts suggest that the Thickened Stochastic Fields approach or an Adaptive Mesh Refinement (AMR) approach may be needed in order to obtain numerically-accurate Stochastic Fields predictions for practical systems.

A known disadvantage of thickened flame approaches is that application of the thickening factor and the efficiency function affects the chemical time scales in the fluid, and consequently changes the Damköhler and Karlovitz numbers describing the turbulence-chemistry interactions. The Damköhler number (Da= *L*_*T*_*s*_*L*_/*u*′*δ*_*L*_) and Karlovitz number (Ka$=(u^{\prime 3}\delta _{L}/{S_{L}^{3}} L_{t})^{1/2}$) change by *E*/*F* and (*F*/*E*^3^)^1/2^ respectively. Since the turbulent diffusivity and micromixing terms in Eq. 6 lead to a stochastic field reaction front thickness that is greater than the corresponding laminar flame thickness by the factor $f_{\delta }=\delta _{c^{*}}/\delta _{L}$ described by Eq. 13, the Thickened Stochastic Fields requires a thickening factor that is smaller than for the conventional Thickened Flame approach by the same factor *f*_*δ*_. In the case of the Bunsen flame LES in this study, the thickening factor is expected to be 1.3-1.7 times less than in a conventional thickened flame LES with the same filter scale. The undesirable effect of thickening on the Damköhler and Karlovitz numbers is therefore generally smaller for the Thickened Stochastic Fields approach compared to the conventional Thickened Flame approach, and the correct chemical time scale and the conventional Stochastic Fields model is recovered in the limit of adequate resolution, for which no thickening is required.

### Bunsen flame analysis

**Fig. 6:**
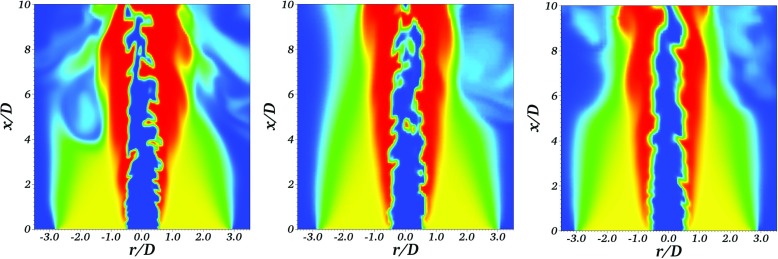
Instantaneous temperature contours of an individual stochastic field from the Bunsen flame LES: Stochastic Fields with Δ_*x*_ = 0.5 mm Δ = 1.0 mm; Stochastic Fields with Δ_*x*_ = Δ = 1.0 mm; Thickened Stochastic Fields with Δ_*x*_ = Δ = 1.0 mm

**Fig. 7:**
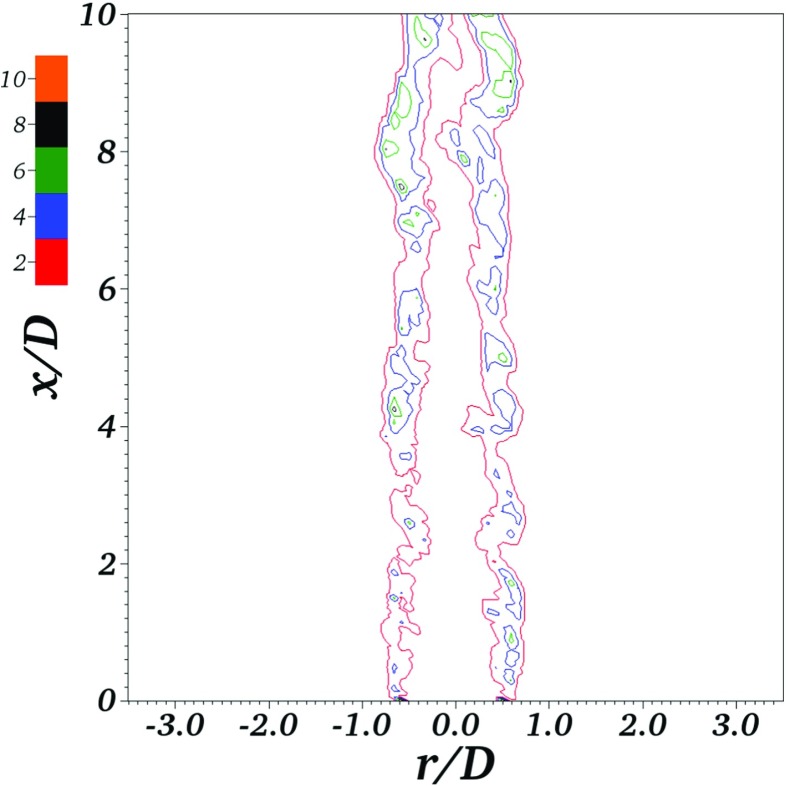
Instantaneous contours of thickening factor in the Thickened Stochastic Fields simulation

**Fig. 8:**
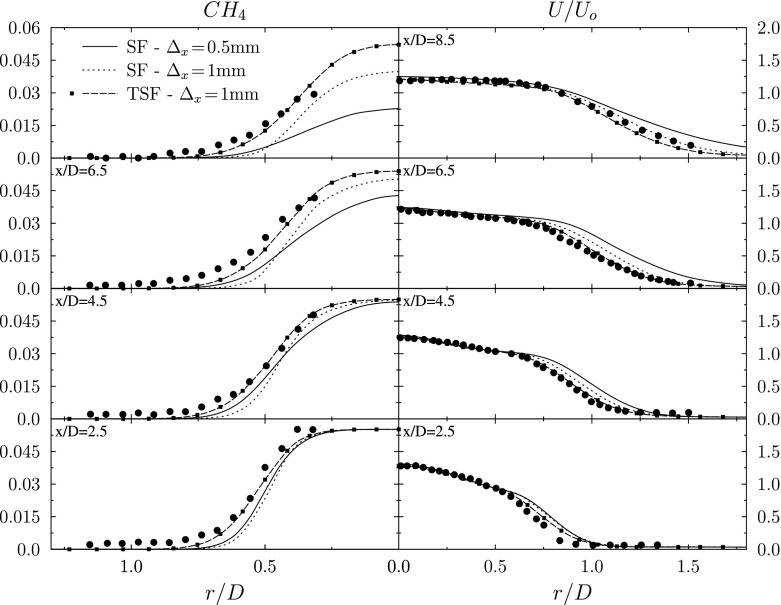
Radial distributions of the time-averaged methane mass fraction $\langle \widetilde {Y_{CH_{4}}}\rangle $ and the normalised mean axial velocity at various axial locations for the three different simulations

Results of three simulations of the F3 Bunsen flame are presented in Figs. 6, 7 and 8. The LES filter length scale Δ is set equal to 1*mm* in all three cases. The first simulation solves the Stochastic Fields equation (Eq. 5) and adopts the conventional practice of setting the grid spacing equal to the filter scale, Δ_*x*_ = Δ = 1*mm*. The second simulation solves Eq. 5 with improved numerical resolution, using a grid characterised by Δ_*x*_ = Δ/2 = 0.5*mm*. The third simulation solves the Thickened Stochastic Fields equation (Eq. 6) with Δ_*x*_ = Δ = 1*mm*.

Figure 6 presents the instantaneous filtered temperature fields for an individual stochastic field in each of the three simulations. The images indicate that the flame thickness increases and the flame wrinkling decreases due to application of the Thickened Stochastic Fields model, and that the flame height increases. Use of “improved resolution” leads to a reduction in flame thickness and increased flame wrinkling. The different flame thicknesses in the three simulations are quantified by evaluating an indicative stochastic fields reaction front thickness given by the inverse average progress variable gradient magnitude on the stochastic fields conditioned on the progress variable giving maximum heat release, 〈|∇*ζ*_*c*,(*i*)_|∣*ζ*_*c*,(*i*)_ = 0.68〉^− 1^. Application of the Thickened Stochastic Fields model leads to the average thickness of the stochastic field reaction fronts increasing from 2.68 mm in the conventional Stochastic Fields simulation to 4.9 mm in the Thickened solution – confirming that the Thickened Stochastic Fields approach delivers the target five grid spacings within the stochastic field reaction fronts. Use of the “improved resolution” reduces the thickness to 1.8 mm, indicating that the numerical solution of the Stochastic Fields equation is not grid-independent with Δ_*x*_ = Δ [4].

A contour map of the thickening factor used in the Thickened Stochastic Fields model is presented in Fig. 7. The thickening factor is unity outside of the flame since the flame sensor tends to zero. Inside the flame, the value of the thickening factor varies due to the dependence on the local value of $u^{\prime }_{{\Delta }}/S_{L}$ in Eq. 13. The peak value of the thickening factor within the flame increases with downstream distance: in the near-field the peak thickening factor is typically in the range 2-4, and it is mostly in the range 2-6 downstream of *x*/*D* = 5. The thickening factor tends to increase downstream because the Karlovitz number and $u^{\prime }_{{\Delta }}/S_{L}$ decay away from the jet inlet, leading to the combustion becoming more flamelet-like and thereby increasing the resolution requirement. These thickening factor values may be compared with the fixed thickening factor *F* = 8 used in Ref. [22] for Thickened Flame simulations of the F3 flame with a similar grid spacing, Δ = Δ_*x*_ = 0.8*mm*. Figure 7 shows that the thickening factor required by the Thickened Stochastic Fields approach can be significantly less than the value required in the Thickened Flame model, reducing unwanted effects of the thickening on the chemical time scale, the Damköhler number and the Karlovitz number.

The radial profiles of the predicted mean methane mass fraction and mean axial velocity are shown for the three simulations in Fig. 8. The mean axial velocity shows reasonable agreement with the experimental data in each case, with predictions of the Thickened Stochastic Fields approach showing the closest agreement. Due to thermal expansion, the mean velocity field is affected by the flame location. The Thickened Stochastic Field predicts a longer flame that is closer to the experimental observations and this leads to a correspondingly better prediction of the mean velocity field.

The methane mass fraction profiles in Fig. 8 show that the two Stochastic Field simulations over-predict the rate of flame propagation, leading to the shorter flame length. The improved resolution case with Δ_*x*_ = 0.5*mm* displays faster flame propagation than the less well-resolved case with Δ_*x*_ = 1.0*mm*. Under-resolution affects the flame speed in three main ways [4]: first, numerical diffusion increases the local propagation speed of the individual stochastic field reaction fronts; second, thickening of the stochastic field reaction fronts caused by the numerical diffusion reduces the amount of flame wrinkling produced by a given velocity field; third, the numerical viscosity reduces the strength of small-scale eddies that wrinkle the reaction fronts. Since the present Bunsen flame exhibits combustion in the flamelet regime, the scales of the reaction fronts are generally smaller than the scales of the velocity field that wrinkles the flame. This suggests that a small degree of under-resolution primarily will affect the reaction front propagation speed, without affecting the flame wrinkling significantly – leading to an over-prediction of flame speed. More significant under-resolution increasingly will tend to affect the amount of flame wrinkling – reducing the predicted flame speed. The present Stochastic Fields LES of the F3 Bunsen flame with a 1 mm filter scale was predicted to require a maximum 0.11-0.14 mm grid spacing in order to resolve the stochastic field reaction fronts fully. This suggests that, with either the 1 mm grid spacing or the “improved resolution” 0.5 mm grid spacing, the present Stochastic Fields simulations remain substantially under-resolved. The observation that the predicted flame speed increases when refining the grid spacing to 0.5 mm indicates that this change in resolution affects the flame speed primarily through an increase in flame wrinkling, thereby taking the predicted flame propagation speed further away from the experimental observations. Further grid refinement beyond Δ_*x*_ = Δ/2 is not expected to have a significant further effect on the strength of eddies in the velocity field [23]. However, since the reaction fronts are expected to be around five times thinner in a fully-resolved Stochastic Fields simulation compared to in the “improved resolution case”, we anticipate that the flame propagation speed may start to reduce towards the speed seen in the experiment as the grid is refined further.

Full-resolution Stochastic Fields simulations however are not currently available. Since the computational effort for a fully-resolved simulation would be (0.5*mm*/0.11*mm*)^3^ = 93 times greater than for the “improved resolution” case, the computational resource for such a computation is prohibitive, even for this laboratory flame configuration. Such fully-resolved Stochastic Fields simulations of premixed turbulent combustion will be facilitated by application of AMR approaches, however introduction of AMR-Stochastic Fields LES is beyond the scope of this study. The Thickened Stochastic Fields approach however accounts for the loss of flame surface area due to the thickening applied in the model, and does not suffer from numerical diffusion effects since the thickened reaction fronts are well-resolved. Overall the Thickened Stochastic Fields prediction is in good agreement with the experiment. The simple addition of thickening terms in the Thickened Stochastic Fields equation thereby yields a computationally-efficient and accurate alternative to full-resolution Stochastic Fields simulations, which remain computationally prohibitive without adoption of AMR techniques.

## Conclusions

A Thickened Stochastic Fields approach is proposed that seeks to ensure adequate numerical resolution of the Stochastic Fields equation in premixed turbulent combustion with reduced computation time. The Thickened Stochastic Fields formulation bridges between the conventional Stochastic Fields and conventional Thickened-Flame approaches depending on the numerical grid spacing utilised. A method for determining for the thickening factor required and an efficiency function model are provided, based on data from one-dimensional Stochastic Fields simulations of freely-propagating turbulent premixed flames. The efficiency function accounts for the loss of resolved flame surface area caused by applying the thickening transformation to the Stochastic Fields equations. The numerical implementation of the Thickened Stochastic Fields approach requires only a minor modification of the Stochastic Fields code. The Thickened Stochastic Fields approach is tested by performing LES of a laboratory Bunsen flame. The results demonstrate that the Thickened Stochastic Fields method avoids numerical errors arising in Stochastic Fields simulations where the grid spacing is set equal to the filter scale and produces results that agree closely with the experimental measurements. The thickening factor required in the Thickened Stochastic Fields approach is generally less than in the conventional Thickened Flame approach, and this promises superior modelling of flame-turbulence interactions compared to the Thickened Flame approach. The Thickened Stochastic Fields approach therefore permits numerically-accurate simulation of industrially-relevant premixed combustion systems with several orders of magnitude fewer grid points than are required for accurate solution of the unthickened Stochastic Fields approach.

## References

[1] Valiño L (1998). A field Monte Carlo formulation for calculating the probability density function of a single scalar in a turbulent flow. Flow Turbul. Combust..

[2] Jones W, Navarro-Martinez S, Röhl O (2007). Large eddy simulation of hydrogen auto-ignition with a probability density function method. Proc. Combust. Inst..

[3] Dodoulas I, Navarro-Martinez S (2013). Large eddy simulation of premixed turbulent flames using the probability density function approach. Flow Turbul. Combust..

[4] Picciani, M.A., Richardson, E.S., Navarro-Martinez, S.: Resolution requirements in stochastic field simulation of turbulent premixed flames. Flow Turbul. Combust. (2018). 10.1007/s10494-018-9953-z 10.1007/s10494-018-9953-z PMC6297202 30613189

[5] Colin O, Ducros F, Veynante D, Poinsot T (2000). A thickened flame model for large eddy simulations of turbulent premixed combustion. Phys. Fluids.

[6] Charlette F, Meneveau C, Veynante D (2002). A power-law flame wrinkling model for LES of premixed turbulent combustion Part I: non-dynamic formulation and initial tests. Combust. Flame.

[7] Yu S, Navarro-Martinez S (2015). Large eddy simulation modelling of flame acceleration, detonation and deflagration to detonation transition. Proc. Combust. Inst..

[8] Valiño L, Mustata R, Ben Letaief K (2016). Consistent behavior of Eulerian Monte Carlo fields at low Reynolds numbers. Flow Turbul. Combust..

[9] Dopazo C, O’Brien EE (1974). An approach to the autoignition of a turbulent mixture. Acta Astronaut..

[10] Prasad N (2011). Large eddy simulation of partially premixed turbulent combustion.

[11] Chen YC, Peters N, Schneemann GA, Wruck N, Renz U, Mansour MS (1996). The detailed flame structure of highly stretched turbulent premixed methaneair flames. Combust. Flame.

[12] Lindstedt RP, Vaos EM (2006). Transported PDF modeling of high-Reynolds-number premixed turbulent flames. Combust. Flame.

[13] Stöllinger M, Heinz S (2008). PDF modeling and simulation of premixed turbulent combustion. Monte Carlo Methods Appl..

[14] Stöllinger M, Heinz S (2010). Evaluation of scalar mixing and time scale models in PDF simulations of a turbulent premixed flame. Combust. Flame.

[15] Yilmaz SL, Nik MB, Givi P, Strakey PA (2010). Scalar filtered density function for large eddy simulation of a bunsen burner. J. Propuls. Power.

[16] Smagorinsky J (1963). General circulation experiments with the primitive equations. Mon. Weather. Rev..

[17] Jones W, di Mare F, Marquis A (2002). LES BOFFIN: Users guide.

[18] Kloeden P, Platen E (1992). Numerical solution of stochastic differential equations.

[19] Sabel’nikov V, Soulard O (2005). Rapidly decorrelating velocity-field model as a tool for solving one-point Fokker-Planck equations for probability density functions of turbulent reactive scalars. Phys. Rev. E Stat. Nonlinear Soft Matter Phys..

[20] Klein M, Sadiki A, Janicka J (2003). A digital filter based generation of inflow data for spatially developing direct numerical or large eddy simulations. J. Comput. Phys..

[21] Durand, L., Polifke, W.: Implementation of the thickened flame model for large eddy simulation of turbulent premixed combustion in a commercial solver. In: ASME Proc., pp 869–878 (2007)

[22] Wang G, Boileau M, Veynante D (2011). Implementation of a dynamic thickened flame model for large eddy simulations of turbulent premixed combustion. Combust. Flame.

[23] Vreman B, Geurts B, Kuerten H (1996). Comparison of numerical schemes in large-eddy simulation of the temporal mixing layer. Int. J. Numer. Methods Fluids.

